# Supplementary material for: Population genomics reveals the origin and asexual evolution of human infective trypanosomes
Source: eLife. 2016 Jan 26;5:e11473. doi: 10.7554/eLife.11473 (PMC4739771; doi:10.7554/eLife.11473)
Supplement: Figure 1—source data 1. — DOI: http://dx.doi.org/10.7554/eLife.11473.004 [file elife-11473-fig1-data1.docx]

## Figure 1 – source data 1. Number of SNP loci with respect to different sub-species

|  | **Tbb**  (n=2) | **Tbg1** (n=75) | **Tbg2** (n=4) | **Tbr** (n=4) | **All sub-species together** (n=85) |
| --- | --- | --- | --- | --- | --- |
| **n of SNP loci vs Tb927** | 122,104 | 130,180 | 160,637 | 169,359 | 230,891 |
| **Density (SNPs/10 Kb)** | 56 | 59 | 73 | 77 | 105 |
| **n of SNP loci within each sub-species** | 122,104 | 11,398 | 55,312 | 95,980 | 230,891 |
| **Density (SNPs/10 Kb)** | 56 | 5 | 25 | 44 | 105 |

Tbb = *T.b. brucei*; Tbg1 = *T.b. gambiense* Group 1, Tbg2 = *T.b. gambiense* Group 2, Tbr = *T.b. brucei rhodesiense*. The number of SNP loci was calculated for each sub-species using two methods, (1) in comparison to the Tb927 reference genome and (2) in comparison to other members of the sub-species. Ten-fold fewer SNPs are found among the 75 *T.b. gambiense* Group 1 isolates (n=11,398) than the two *T.b. brucei* isolates (n=122,104) analysed.
