# Supplementary material for: Population genomics reveals the origin and asexual evolution of human infective trypanosomes
Source: eLife. 2016 Jan 26;5:e11473. doi: 10.7554/eLife.11473 (PMC4739771; doi:10.7554/eLife.11473)
Supplement: Figure 1—source data 2. — DOI: http://dx.doi.org/10.7554/eLife.11473.005 [file elife-11473-fig1-data2.docx]

## Figure 1 – source data 2. Testing Hardy-Weinberg Equilibrium across the *T.b. gambiense* Group 1 genome

|  |  |  | SNP loci with P<0.001 | |
| --- | --- | --- | --- | --- |
| Population analysed | **n isolates** | **n polymorphic sites** | **n significant loci** | **% with heterozygote excess** |
| All Tbg1 | 75 | 1,690 | 1,625 (97.4%) | 100% |
| Bonon | 14 | 1,631 | 1,573 (96.4%) | 100% |
| Boffa | 18 | 1,661 | 1,637 (98.5%) | 100% |
| Dubreka | 19 | 1,648 | 1,586 (96.2%) | 100% |

Departure from Hardy-Weinberg Equilibrium (HWE) was assessed using the χ² test for SNP loci across the *T.b. gambiense* Group 1 genome utilising the set of ‘ancient’ SNP loci (Figure 1 – figure supplement 7). Statistically significant loci at P<0.001 were additionally tested to determine whether deviation from HWE was associated with a heterozygote excess. Along with the entire set of *T.b. gambiense* Group 1 isolates (All Tbg1), three spatio-temporally defined sub-populations were tested, namely Bonon in the Côte d'Ivoire and Boffa and Dubreka in Guinea.
