# Supplementary material for: Population genomics reveals the origin and asexual evolution of human infective trypanosomes
Source: eLife. 2016 Jan 26;5:e11473. doi: 10.7554/eLife.11473 (PMC4739771; doi:10.7554/eLife.11473)
Supplement: Figure 1—source data 3. — DOI: http://dx.doi.org/10.7554/eLife.11473.006 [file elife-11473-fig1-data3.docx]

## Figure 1 – source data 3. F_IS_ by sub-population

| Population analysed | n isolates | F_IS_ median | F_IS_ average | F_IS_ std error | Proportion of loci with F_IS_ = -1 | Proportion of loci with low F_IS_ |
| --- | --- | --- | --- | --- | --- | --- |
| Bonon | 14 | -1 | -0.971 | 0.004 | 95.9% | 84.8% |
| Boffa | 18 | -1 | -0.981 | 0.004 | 97.6% | 98.2% |
| Dubreka | 19 | -1 | -0.984 | 0.003 | 97.4% | 98.4% |

F_IS_ was calculated across the *T.b. gambiense* Group 1 genome utilising the set of ‘ancient’ SNP loci (Figure 1 – figure supplement 7). A median figure of -1 was calculated for the entire set of *T.b. gambiense* Group 1 isolates (All Tbg1) and for each of the Bonon, Boffa and Dubreka sub-populations. The proportion of SNP loci exhibiting a statistically significant lower than expected F_IS_ (P<0.001) was calculated using a randomisation method (n iterations = 30,000)^38^.
