# Supplementary material for: Population genomics reveals the origin and asexual evolution of human infective trypanosomes
Source: eLife. 2016 Jan 26;5:e11473. doi: 10.7554/eLife.11473 (PMC4739771; doi:10.7554/eLife.11473)
Supplement: Figure 1—source data 4. — DOI: http://dx.doi.org/10.7554/eLife.11473.007 [file elife-11473-fig1-data4.docx]

## Figure 1 – source data 4. Number and type of *T.b. gambiense* Group 1 SNPs

| **Chromosome** | **Tbg1 SNPs** | **non-LOH** | **MAF>20% non-LOH** | **Tbg1 derived**  **(non-LOH)** | **Tbg1 ancient**  **(non-LOH)** |
| --- | --- | --- | --- | --- | --- |
| **1** | 319 | 46 | 37 | 12 | 19 |
| **2** | 323 | 124 | 94 | 64 | 56 |
| **3** | 638 | 68 | 48 | 26 | 15 |
| **4** | 659 | 189 | 127 | 68 | 88 |
| **5** | 779 | 698 | 533 | 208 | 248 |
| **6** | 663 | 486 | 306 | 206 | 132 |
| **7** | 951 | 844 | 528 | 356 | 258 |
| **8** | 1,249 | 867 | 554 | 349 | 235 |
| **9** | 599 | 30 | 16 | 15 | 11 |
| **10** | 2,441 | 278 | 212 | 76 | 122 |
| **11** | 2,777 | 1,571 | 1,094 | 572 | 506 |
| **Overall** | **11,398** | **5,201** | **3,549** | **1,952** | **1,690** |

A total of 11,398 SNP loci were identified among the 75 isolates of *T.b. gambiense* Group 1. We analysed the data both with and without regions where a Loss of Heterozygosity (LOH)/gene conversion event had occurred. A set of SNP loci was identified which was specific to the *T.b. gambiense* Group 1 population across non-LOH regions of the genome (i.e. Tbg1 derived, n=1,952). Similarly, a set of SNPs loci polymorphic both within and outside the Group 1 population (i.e. ancient) was also defined (n=1,690). Tbg1 = *T.b. gambiense* Group 1, non-LOH = non Loss of Heterozygosity regions.
