# Supplementary material for: Population genomics reveals the origin and asexual evolution of human infective trypanosomes
Source: eLife. 2016 Jan 26;5:e11473. doi: 10.7554/eLife.11473 (PMC4739771; doi:10.7554/eLife.11473)
Supplement: Figure 3—source data 1. — DOI: http://dx.doi.org/10.7554/eLife.11473.015 [file elife-11473-fig3-data1.docx]

## Figure 3 – source data 1. Estimated time since the most recent common ancestor

| **Dataset used to estimate mutation rate** | **Mutation rate (substitutions per base per year)** | **Time to MRCA based on Chromosome 9 LOH region (all SNPs)** | **Time to MRCA based on whole genome SNP set (n=1,952)** |
| --- | --- | --- | --- |
| Present study (using all isolates) | 1.82 x 10^-8^ | 1,325 (+/-88) | 1,258 (+/-39) |
| Present study (using single clade) | 2.09 x 10^-8^ | 1,154 (+/-77) | 1,096 (+/-34) |
| *T. cruzi* GPI (95% CI) | 2.56 x 10^-9^  4.60 x 10^-9^ | 9,420 (+/-628)  5,243 (+/-350) | 8,947 (+/-277)  4,979 (+/-154) |
| *T. cruzi* COII-ND1 (95% CI) | 1.05 x 10^-8^  3.04 x 10^-8^ | 2,297 (+/-153)  793 (+/-53) | 2,181 (+/-67)  753 (+/-23) |

Across the non-LOH part of the genome (8.47 Mb), each isolate acquired, on average, 388 novel mutations (+/12) since the common ancestor of all the samples used in the present study. An alternative method was used whereby the number of accumulated mutations in an ancient LOH region on Chromosome 9, which is conserved in every Group 1 isolate, was estimated. The absence of an abundance of fixed heterozygous loci (i.e. pre-existing, ancestral mutations), allowed us to easily identify novel mutations. It was found that across a 1.55 Mb region of Chromosome 9, 75 (+/-5) accumulated mutations were identified on each genotype. As all the samples were time-stamped, the annual mutation rate could be calculated and this was done using two methods; the first was calculated over all seventy-five isolates while the second utilised a subset of samples representing a discrete lineage isolated over the course of a few years. The additional two rates refer to published mutation rates in *Trypanosoma cruzi*; these represent the house-keeping gene glucose-6-isomerase (*GPI*) and an area spanning the cytochrome oxidase subunit II (*COII*) and NADH dehydrogenase subunit 1 (*ND1*) genes^23^. For each mutation rate and method of estimating the number of mutations, the time to most recent common ancestor (MRCA) was calculated.
