# Supplementary material for: Population genomics reveals the origin and asexual evolution of human infective trypanosomes
Source: eLife. 2016 Jan 26;5:e11473. doi: 10.7554/eLife.11473 (PMC4739771; doi:10.7554/eLife.11473)
Supplement: Figure 4—source data 1. — DOI: http://dx.doi.org/10.7554/eLife.11473.017 [file elife-11473-fig4-data1.docx]

## Figure 4 – source data 1. Co-phylogenetic analysis

| **Chromosome** | **Cost of best-fit solution** | **Random tip mapping P value** | **Random tree P value** |
| --- | --- | --- | --- |
| **1** | 120 | <0.001 | <0.001 |
| **2** | 121 | <0.001 | <0.001 |
| **3** | 104 | <0.001 | <0.001 |
| **4** | 104 | <0.001 | <0.001 |
| **5** | 118 | <0.001 | <0.001 |
| **6** | 106 | <0.001 | <0.001 |
| **7** | 113 | <0.001 | <0.001 |
| **8** | 99 | <0.001 | <0.001 |
| **9** | 101 | <0.001 | <0.001 |
| **10** | 93 | <0.001 | <0.001 |
| **11** | 74 | <0.001 | <0.001 |

Co-phylogenetic analysis was undertaken using the A and B haplotype trees for all 75 *T.b.gambiense* Group 1 isolates across each of the eleven chromosomes (Figure 4 – figure supplement 1). The best-fit solution resolving the topologies of the two trees was compared to the simulated datasets (i.e. ‘random tip-mapping’ and ‘random tree’) and for every chromosome the A and the B haplotype trees were found to be statistically more similar to one another than would be expected by chance.
