# Supplementary material for: Population genomics reveals the origin and asexual evolution of human infective trypanosomes
Source: eLife. 2016 Jan 26;5:e11473. doi: 10.7554/eLife.11473 (PMC4739771; doi:10.7554/eLife.11473)
Supplement: Supplementary file 1. — For each isolate, the year of isolation, host, country and location are given along with the results of the BIIT test (Blood Incubation Infectivity Test), which determines human infectivity. The presence/absence of TgSGP, the T.b. gambiense Group 1 human serum resistance gene and SRA, the T.b. rhodesiense human serum resistance gene are indicated. The majority of samples in this study were T.b. gambiense Group 1, details of which have been previously published (Heitman, 2006; Thorvaldsdottir et al., 2013). DOI: http://dx.doi.org/10.7554/eLife.11473.021 [file elife-11473-supp1.pdf]

Supplementary File 1. Isolates used in this study

T.b. gambiense Group 1

ID	Sample ID	Also known as...	Year	Host	Country	Locality	BIIT test	TgSGP PCR	SRA
1	BIM_1	MHOM/CM/75/BIM	1975	man	Cameroon	Campo	HSR	+	-
2	MOS	MHOM/CM/74/MOS	1974	man	Cameroon	Mbam	HSR	+	-
3	B4_4163P		2004	man	Côte d'Ivoire	Bonon	HSR	+	-
4	B4_F303P		2004	man	Côte d'Ivoire	Bonon	HSR	+	-
5	B4_G2P		2004	man	Côte d'Ivoire	Bonon	HSR	+	-
6	B4_I314P		2004	man	Côte d'Ivoire	Bonon	HSR	+	-
7	CB5_1		2002	man	Côte d'Ivoire	Bonon	HSR	+	-
8	S1_1_6R		2002	man	Côte d'Ivoire	Bonon	HSR	+	-
9	S14_5_1	MHOM/CI/02/S14/5/1	2002	man	Côte d'Ivoire	Bonon	HSR	+	-
10	S15_1_KIVI		2002	man	Côte d'Ivoire	Bonon	HSR	+	-
11	S27_16_13		2000	man	Côte d'Ivoire	Bonon	HSR	+	-
12	S27_2_6		2000	man	Côte d'Ivoire	Bonon	HSR	+	-
13	S3_4_1		2000	man	Côte d'Ivoire	Bonon	HSR	+	-
14	S7_2_2		2002	man	Côte d'Ivoire	Bonon	HSR	+	-
15	T33_1_7_KIVI		2000	man	Côte d'Ivoire	Bonon	HSR	+	-
16	T66_4_2		2000	man	Côte d'Ivoire	Bonon	HSR	+	-
17	DEOLA	MHOM/CI/84/DAL629	1984	man	Côte d'Ivoire	Daloa	HSR	+	-
18	ISTI	MHOM/CI/83/DAL607	1983	man	Côte d'Ivoire	Daloa	HSR	+	-
19	LIGO	MHOM/CI/84/DAL655	1984	man	Côte d'Ivoire	Daloa	HSR	+	-
20	LISA	MHOM/CI/83/DAL642	1983	man	Côte d'Ivoire	Daloa	HSR	+	-
21	SAKON	MHOM/CI/83/DAL403	1983	man	Côte d'Ivoire	Daloa	HSR	+	-
22	SEVAL	MHOM/CI/84/DAL633	1984	man	Côte d'Ivoire	Daloa	HSR	+	-
23	TOBO	DAL596	1983	man	Côte d'Ivoire	Daloa	HSR	+	-
24	ZENOU	MHOM/CI/83/DAL625	1983	man	Côte d'Ivoire	Daloa	HSR	+	-
25	KIDE	MHOM/CI/83/DAL595	1983	man	Côte d'Ivoire	Gagnoa	HSR	+	-
26	T21_4A		1999	man	Côte d'Ivoire	Grand-Zathry	HSR	+	-
27	B7_2		2001	man	Côte d'Ivoire	Oumé	HSR	+	-
28	N1317_2_KIVI		2001	man	Côte d'Ivoire	Oumé	HSR	+	-
29	T4_2_KIVI		2001	man	Côte d'Ivoire	Oumé	HSR	+	-
30	AMAN_KIVI		2001	man	Côte d'Ivoire	Oumé	HSR	+	-
31	CP1_2_KIVI		2001	man	Côte d'Ivoire	Oumé	HSR	+	-
32	YAD		1998	man	Côte d'Ivoire	Sinfra	HSR	+	-
33	N2561		1997	man	Côte d'Ivoire	Sinfra	HSR	+	-
34	ELIANE	MHOM/CI/58/ELIANE	1952	man	Côte d'Ivoire	Unknown	HSR	+	-
35	ABTOR	MHOM/CI/83/DAL543	1983	man	Côte d'Ivoire	Vavoua	HSR	+	-
36	ADZAM	MHOM/CI/83/DAL542	1983	man	Côte d'Ivoire	Vavoua	HSR	+	-
37	BRAZO	MHOM/CI/84/DAL740	1984	man	Côte d'Ivoire	Vavoua	HSR	+	-
38	SETRA	MHOM/CI/79/THDAL083	1979	man	Côte d'Ivoire	Vavoua	HSR	+	-
39	DOB112_KIVI		2002	man	Guinea	Boffa	HSR	+	-
40	DOB7_1		2002	man	Guinea	Boffa	HSR	+	-
41	LAB13_5		2002	man	Guinea	Boffa	HSR	+	-
42	LAB15_2		2002	man	Guinea	Boffa	HSR	+	-
43	LAB27_4		2002	man	Guinea	Boffa	HSR	+	-
44	LAC11_1		2002	man	Guinea	Boffa	HSR	+	-

	Sample ID	Also known as…	Year	Host	Country	Locality	BIIT test	TgSGP PCR	SRA
45	LAC14_3		2002	man	Guinea	Boffa	HSR	+	-
46	LAC22_11		2002	man	Guinea	Boffa	HSR	+	-
47	SAC23_3		2002	man	Guinea	Boffa	HSR	+	-
48	TH1_29_7		2002	man	Guinea	Boffa	HSR	+	-
49	WAB18_23		2002	man	Guinea	Boffa	HSR	+	-
50	WAB22_6		2002	man	Guinea	Boffa	HSR	+	-
51	WAB6_1		2002	man	Guinea	Boffa	HSR	+	-
52	YEMB3_7		2002	man	Guinea	Boffa	HSR	+	-
53	LAC22_10		2002	man	Guinea	Boffa	HSR	+	-
54	SAC1_2		2002	man	Guinea	Boffa	HSR	+	-
55	YEMB41_2		2002	man	Guinea	Boffa	HSR	+	-
56	YENB17_4		2002	man	Guinea	Boffa	HSR	+	-
57	B13_9		1998	man	Guinea	Dubreka	HSR	+	-
58	B15_7		1998	man	Guinea	Dubreka	HSR	+	-
59	B34_2		1998	man	Guinea	Dubreka	HSR	+	-
60	B5_2		1998	man	Guinea	Dubreka	HSR	+	-
61	F10_5		1998	man	Guinea	Dubreka	HSR	+	-
62	F12_20		1998	man	Guinea	Dubreka	HSR	+	-
63	F31_4		1998	man	Guinea	Dubreka	HSR	+	-
64	F34_1		1998	man	Guinea	Dubreka	HSR	+	-
65	F35_2		1998	man	Guinea	Dubreka	HSR	+	-
66	F4_1		1998	man	Guinea	Dubreka	HSR	+	-
67	F55_3		1998	man	Guinea	Dubreka	HSR	+	-
68	F7_6		1998	man	Guinea	Dubreka	HSR	+	-
69	KAC4_10		2002	man	Guinea	Dubreka	HSR	+	-
70	KHOB34_1		2002	man	Guinea	Dubreka	HSR	+	-
71	N4ANNEE_13_KIVI		2002	man	Guinea	Dubreka	HSR	+	-
72	N70_2		1998	man	Guinea	Dubreka	HSR	+	-
73	BROB7_16_KIVI		2002	man	Guinea	Dubreka	HSR	+	-
74	F2_1		1998	man	Guinea	Dubreka	HSR	+	-
75	KEMB9_5		2002	man	Guinea	Dubreka	HSR	+	-

T.b. gambiense Group 2

ID	Sample ID	Also known as...	Year	Host	Country	Locus	BIIT test	TgSGP PCR	SRA
76	TSW33		1982	man	Côte d'Ivoire	Bouafle	VHSR	-	-
77	KOBIR	MHOM/CI/82/DAL503	1982	man	Côte d'Ivoire	Daloa	VHSR	-	-
78	OUSOU	MHOM/CI/82/THDAL494	1982	man	Côte d'Ivoire	Daloa	VHSR	-	-
79	TB386	MHOM/CI/78/TH114	1978	man	Côte d'Ivoire	Koudougou	VHSR	-	-

T.b. rhodesiense

	Sample ID	Also known as...	Year	Host	Country	Locus	BIIT test	TgSGP PCR	SRA
80	K975		1970	Fly	Kenya	Kiboko	VHSR	-	+
81	N1052		1961	man	Kenya	Nyanza	VHSR	-	+
82	GYBO		1984	man	Tanzania	Tanzania	VHSR	-	+
83	Z210		1982	man	Zambia	Luangwa	VHSR	-	+

T.b. brucei

	Sample ID	Also known as...	Year	Host	Country	Locus	BIIT test	TgSGP PCR	SRA
84	TB927	TREU927/4	1969	Fly	Kenya	Unknown	- PCR	-	-
85	TB247	STIB247	1971	Hartebe est	Tanzania	Serengeti	HSS	-	-

For each isolate, the year of isolation, host, country and location are given along with the results of the BIIT test (Blood Incubation Infectivity Test), which determines human infectivity. The presence/absence of *TgSGP*, the *T.b. gambiense* Group 1 human serum resistance gene and *SRA*, the *T.b. rhodesiense* human serum resistance gene are indicated. The majority of samples in this study were *T.b. gambiense* Group 1, details of which have been previously published[2,46].
